# Validation of the Arabic version of the smoking cessation motivation questionnaire among Tunisian student smokers

**DOI:** 10.12688/f1000research.167614.1

**Published:** 2025-10-14

**Authors:** Selma Gallas, Hela Ghali, Houyem Said Latiri

**Affiliations:** 1 Medical Intensive Care Unit, Farhat Hached University Hospital, University of Sousse, Faculty of Medicine of Sousse, Sousse, Tunisia; 2 Department of Preventive and Community Medicine, Sahloul University Hospital, University of Sousse, Faculty of Medicine of Sousse, Sousse, Tunisia

**Keywords:** Psychometrics, Translations, Smoking Cessation, Surveys and Questionnaires, Motivation, Students, Smokers

## Abstract

**Background:**

Smoking remains a major public health problem, particularly among Tunisian health students, with a prevalence of 26%. Assessing motivation to quit smoking requires tools that have been validated in the local language. This study aimed to translate and validate the psychometric properties of the Motivation to Quit Smoking Questionnaire (Q-MAT) into Arabic.

**Method:**

A cross-sectional methodological study was conducted among 203 smoking health science students in March 2022. The cross-cultural validation process followed Vallerand’s seven steps, including translation/back-translation, expert panel review, pre-testing, and psychometric analyses. Reliability was assessed by internal consistency (Cronbach’s alpha) and temporal stability (test-retest). Content validity was measured by the CVI index, and construct validity by exploratory factor analysis.

**Results:**

The sample included 197 students (mean age: 18.95±1.07 years, sex ratio: 0.89). The Arabic Q-MAT demonstrated excellent reliability with a Cronbach’s alpha of 0.840, a test-retest correlation of 0.831, and an intraclass correlation coefficient of 0.886. Content validity was satisfactory (CVI = 0.89). Principal component analysis revealed that two factors explained 89.148% of the total variance.

**Conclusions:**

This initial validation of the Q-MAT in literary Arabic provides a reliable and valid tool for assessing motivation to quit smoking among Arabic-speaking populations, facilitating the development of targeted public health interventions.

## Background

Smoking is a behavior, reinforced by both physical and psychological dependence. This dependence is mainly due to the addictive effect of nicotine, a psychoactive substance found in tobacco. Physical dependence manifests itself in withdrawal symptoms when smoking is stopped, while psychological dependence is linked to emotional and behavioral factors such as anxiety, stress and social habits. Despite awareness campaigns and public health efforts, smoking remains a major health problem worldwide ( Adouard et al., 2022). And among the various complicating factors is a low level of motivation to change on the part of smokers ( Ghali et al., 2019). According to a study carried out in France in 2020, the overall prevalence of smoking was 17.8% , ranging from 15.8% in medicine to 21.8% in nursing. Nursing students were significantly more frequent smokers than other health students (p < 0.001) ( Pougnet et al., 2021).

In Tunisia, according to a study carried out at the Tunis Faculty of Medicine, overall smoking prevalence was 26% among 291 medical students, with a sex ratio equal to 0.48 and a female predominance ( Ezzaouia et al., 2020). In another study of 440 students at private and state nursing institutes (Tunisia), the sex ratio was 0.65 and smoking prevalence was 20.6%. It was significantly higher in men than in women (50% versus 4.5%; p < 10-3) ( Ben Rejeb et al., 2016). The smoking epidemic in Tunisia remains a major public health problem. It is essential to raise awareness among future doctors and nurses of their vital role in preventing and changing smoking behavior ( Ezzaouia et al., 2020).

Then, according to a study carried out in Tunisia over a five-year period 2015-2020, the probability of quitting smoking was higher among male study participants (p=0.004, OR=9.708) with a high quit motivation score (p=0.001, OR=1.980) ( Mziou et al., 2024). In another Tunisian study carried out over the period 2015-2020, among 93 participants who smoked, the prevalence of smoking cessation was 54.8% (n = 51), 44.1% (n = 41), 35.5% (n = 33), 31.2% (n = 29) and 24.7% (n = 23) at one week, 1 month, 3 months, 6 months and 12 months respectively ( Ghali et al., 2022).

Interventions aimed at reducing smoking prevalence among health students could be significantly enhanced by incorporating components that address perceptions of smoking-related risks. A deeper understanding of how these perceptions influence behavior is crucial for developing effective strategies. Several theoretical frameworks have been proposed to explain and influence health-related behaviors, each offering unique insights into the mechanisms of change. Among these, Prochaska and DiClemente’s trans-theoretical model ( 1986) provides a valuable perspective by framing behavioral change as a dynamic, evolving process. This model categorizes individuals according to their stage of change, ranging from pre-contemplation to maintenance, and helps to identify their readiness and propensity to engage in smoking cessation efforts. By leveraging such theories, tailored interventions can better address the psychological and behavioral dimensions of smoking cessation, particularly within the context of health students who may face unique pressures and influences.

So, integrating these changes can be a long and arduous process, as smokers’ behaviors is dependent on their previous beliefs and attitudes towards this addiction ( Mourre & Gurviez, 2015). Motivation is widely recognized as one of the primary determinants of success in smoking cessation efforts. While various definitions of motivation have been proposed, this study adopts the definition provided by the Philosophy Studies Council in the USA: “The probability that an individual will adhere to, commit to, and pursue a specific action for change” ( Buczkowski et al., 2014). The assessment of motivation to quit smoking relies on validated measurement instruments, which are primarily available in English and French. One such instrument was developed and validated by Aubin et al. (2004) in the context of a French-speaking population. However, there is a notable gap in the availability of equivalent tools for Arabic-speaking populations. Developing and validating an Arabic version of this instrument is essential to meet clinical and research needs, ensuring that motivation to quit smoking is effectively assessed across diverse linguistic and cultural groups.

The aim of our study was to translate and evaluate the psychometric properties of the Smoking cessation motivation questionnaire (Q-MAT) in its Arabic version.

## Materials and methods

### Research design

We conducted a methodological cross-sectional study aimed at evaluating the psychometric properties of the Q-MAT (Smoking Cessation Motivation Questionnaire) using a cross-cultural validation framework. Our approach followed a comprehensive seven-step transcultural validation process including: (1) forward and back-translation by independent bilingual translators, (2) expert panel review for semantic, idiomatic, experiential, and conceptual equivalence, (3) pre-testing with cognitive interviewing among 40 participants, (4) content validity assessment using Content Validity Index with 4-point relevance scale, (5) reliability analysis through internal consistency (Cronbach’s alpha) and test-retest correlation for temporal stability, (6) construct validity through exploratory factor analysis using principal component analysis, and (7) norm establishment through statistical indices calculation ( Vallerand, 1989).

### Study setting

Data were collected in March, 2022, in the academic institutions that agreed to participate in the study, higher institute of nursing, Faculty of Medicine, and private nursing care institute to reach a more heterogeneous population.

### Participants

Participants were first-year health science students of both genders who consented to participate and were classified based on self-reported tobacco use as follows: Light smokers who reported smoking from one to ten cigarettes a day; moderate smokers who reported smoking from 11 to 19 cigarettes a day; and heavy smokers who reported smoking at least 20 cigarettes.

### Data collection

The self-administered questionnaire included two sections: Sociodemographic and smoking-related data: age, gender, marital status, socioeconomic level, housing status, age at first cigarette, daily cigarette consumption, intention to quit or reduce smoking, and parental smoking history. And, Q-MAT scale; a 4-item tool that assesses motivation to quit smoking. Each item is scored, and the total score (0–20) classifies motivation into three levels ( Aubin et al., 2004): <6: low motivation; 7–13: moderate motivation; 13: high motivation.

### Translation and validation process

In our study, the Q-MAT translation and validation process lasted 12 months, from March 28, 2021 to March 28, 2022. The method involved seven stages ( Haoues et al., 2021):

1) Translation and Back-translation: Two independent bilingual translators translated the original French version into Arabic. Two other translators, blinded to the original version, back-translated the Arabic version into French. Discrepancies were reviewed and resolved by the research team.
2) Expert Panel Review: A panel of four experts in health psychology, psychometrics, and public health reviewed all versions and developed a pre-final version. The evaluation followed Vallerand’s (1989) framework for semantic, idiomatic, experiential, and conceptual equivalence ( Vallerand, 1989).
3) Pre-testing and Cognitive Interviewing: Expert consensus were reached through a modified Delphi process involving two rounds of evaluation. In the first round, each expert independently rated item clarity and relevance on a 4-point scale. Items with disagreement were revised and re-evaluated in a second round until consensus (≥80% agreement) was achieved. The pre-final version was subsequently piloted among 40 Tunisian health students who smoke, with feedback collected on clarity and comprehension and additional revisions made using the same Delphi consensus approach ( Steurer, 2011).
4) Content and Concurrent Validity: A panel of experts assessed content validity using the Content Validity Index (CVI), rating each item on a 4-point relevance scale (1 = not relevant to 4 = highly relevant). A CVI of ≥0.80 was considered acceptable ( Waltz et al., 2017). Concurrent validity was also explored using bilingual participants to compare the original and translated versions.
5) Reliability Analysis: Internal consistency was assessed via Cronbach’s alpha. The test–retest reliability was evaluated by administering the Q-MAT twice, one month apart, to the same group of students. The intraclass correlation coefficient (ICC) was calculated with a 95% confidence interval to assess temporal stability ( Vallerand, 1989).
6) Construct Validity: Exploratory factor analysis using principal component analysis was conducted to examine the underlying structure of the Q-MAT. Suitability of data for factor analysis was confirmed using the Kaiser-Meyer-Olkin (KMO) measure (>0.5) and Bartlett’s test of sphericity (p < 0.05) ( Vallerand, 1989).
7) Establishing norms through population selection and statistical indices: Norms for the Arabic version were established by calculating means, standard deviations, percentiles, and Z- and T-scores for the total Q-MAT score, allowing future comparative use in clinical and research settings ( Vallerand, 1989).

### Statistical analysis

Statistical analyses were performed using IBM SPSS Statistics version 25.0 (IBM Corp., Armonk, NY, USA). Normality was assessed using Shapiro-Wilk test. Categorical variables were reported as frequencies and percentages, while continuous variables were summarized using mean and standard deviation (SD) for normally distributed data, or median and interquartile range otherwise.

For reliability assessment, Cronbach’s alpha was calculated with 95% confidence intervals, with values ≥0.70 considered acceptable internal consistency. Test-retest reliability was evaluated using Pearson correlation coefficient and intraclass correlation coefficient (ICC) with 95% CI using a two-way mixed model for absolute agreement.

For validity assessment, content validity was evaluated with a CVI ≥0.80 considered acceptable. For exploratory factor analysis, data adequacy was confirmed using Kaiser-Meyer-Olkin (KMO) measure >0.50 and Bartlett’s test of sphericity (p < 0.05). Principal component analysis was conducted using eigenvalue >1.0 criterion and scree plot examination for factor retention.

Finally, norms for the Arabic version were established by calculating means, standard deviations, percentiles, and Z- and T-scores for the total Q-MAT score.

### Ethical considerations

The study protocol was registered with the Pan African Clinical Trial Registry (PACTR202411479266920) and received ethical approval from the Ethics Committee of Faculty of Medicine of Sousse, Tunisia (registration number N°112, ref: CEFMS 112/2022). Authorizations have also been obtained from the directors of the study sites. Written informed consent was obtained from all participants prior to their enrollment in the study. Although the participants were students at their university, they were all of legal age (over 18 years old) and were informed that anonymity and confidentiality of data were guaranteed and, therefore, no parental consent or assent procedures were required. And participants were explicitly informed during the consent process that their individual data would not be made publicly available and that access would be restricted to the research team to protect their privacy and confidentiality.

## Results

### Demographic characteristics of participants

203 student smokers were included. Their mean age was 18.95±1.07 years with extremes of 18-27 years. The sex ratio was 0.89 and 53.7% of participants had a fairly satisfactory socioeconomic status, 73.9% lived in an urban environment with a family (18.2%) and living parents (54.2%) ( Table 1).

**Table 1.** Characteristics of health student smoker within the city of Sousse, Eastern Tunisia (n=203).

Variable	Number	Relative frequency, %
Gender		
Male	96	47.3
Female	107	52.7
Age, years		
Mean (SD)	18.95±1.07 years	
Socioeconomic status		
Unsatisfactory	50	24.6
Fairly satisfactory	109	53.7
Satisfactory	59	21.7
Environment		
Urban	150	73.9
Rural	53	26.1
Family situation		
Parents living together	110	54.2
Divorced parents	62	30.5
Orphan	31	15.3
Place of residence		
WIth family	37	18.2
In a university hostel	56	27.6
In a shared apartment	59	29.1
Individual rental	48	23.6
With a relative	3	1.5
The average age of the first cigarette	17.08±1.51 years	
The average number of cigarettes smoked per day	13.71±7.81 cigarettes	
Tried to cut down or quit smoking	55	27.1
Had a father smoker	68	33.5
Had a mother smoker	53	26.1

### Tobacco consumption data

The average age of the first cigarette was 17.08±1.51 years, with an average number of cigarettes smoked per day of 13.71±7.81. Only 27.1% of participants had tried to cut down or quit smoking, knowing that 33.5% had a father who smoked and 26.1% had a mother who smoked ( Table 1).

### Fidelity of the experimental version of the Q-MAT

The sample of 203 student smokers who agreed to answer the questionnaire and attend the study were invited to answer the experimental version of the Q-MAT twice, one month apart, by sending them e-mails. 197 participants completed the questionnaire a second time, for a response rate of 48.09%. Test-retest correlation coefficients for the overall Q-MAT score showed satisfactory temporal stability at one month (0.831) ( Table 2). Means and standard deviations of the global score were similar between test 11.43±3.34 and retest 10.46±2.96 ( Table 2). The intra-class correlation coefficient value was 0.886; close to 1, indicating similarity of responses within the same group over the time interval ( Table 2). And internal consistency, assessed by Cronbach s alpha, was 0.840.

**Table 2.** Test-retest of the experimental version (n=197).

	Score in test	Score in retest
Score	M (SD)	M (SD)
Global score	11.43(3.34)	10.46(2.96)
ICC: intra-class correlations coefficient	0.886	
Satisfactory temporal stability at one month	0.831	

### Validity of the experimental version

**Content validity:** The validity of the modified sections of the experimental questionnaire was evaluated by a panel of four experts, who assessed the relevance and accuracy of each item in relation to the construct being measured. The Content Validity Index (CVI) obtained for the instrument was 0.84, indicating that the items were appropriately formulated to reflect the concepts under investigation.

**Construct validity:** The analysis, based on the responses of 197 student smokers to the questionnaire, produced satisfactory results. We can observe that all the questionnaire items appear to be correlated ( Table 3), with the results of the Kayser-Mayer-Olkin tests (0.711) indicating that the correlations between the items are of good quality. And the result of Bartlett’s sphericity test is significant (p < 10 ^-3^).

**Table 3.** Principal component analysis, n=197.

	Total	% of variance	% cumulative
Q-Mat 1	4.514	64.484	64.484
Q-Mat 2	1.726	24.664	89.148
Q-Mat 3	0.382	5.452	94.600
Q-Mat 4	0.220	3.144	97.744
Q-Mat 5	0.103	1.471	99.214
Q-Mat 6	0.043	0.613	99.827
Q-Mat 7	0.012	0.173	100.000
**KMO index and Bartlett test**			
KMO index	0.711		
Bartlett test	p < 10 ^−3^		

Then, principal Component Analysis is a statistical method often used in the validation of scales, to check whether the questions or items of the scale actually measure the concepts they are supposed to measure. After analyzing the results, we see that item 1 of the Q-Mat scale is a component that explains 64.484% of the total variance, which is a significant share. And item 2 adds 24.664% of the variance explained, bringing the total to 89.148%. So, the first two components together explain 89.148% of the total variance, which is very high. This suggests that most of the information in the data can be captured by these two components from a Tunisian health student population. And the other components have eigenvalues well below 1, suggesting that they do not explain much additional variance and may not be very significant in interpreting the data.

And, the group of experts then met again to validate the content of the questionnaire. The recalculated Content Validity Index was 0.89, which made it possible to determine the latest Arabic translation of the questionnaire.

**Q-MAT norm setting:** in our study, the Q-MAT Z and T scores showed Gaussian curves: The overall Q-MAT score also had a Gaussian distribution (Z (-0.157; 1.13); T (11.23; 49.53).

## Discussion

This survey was conducted among 197 smoking health students to translate and analyze the psychometric properties of the Q-MAT literary Arabic version. The results showed that psychometric validation according to Vallerand’s cross-cultural validation produced a reliable and valid Arabic version.

### Preparation of the preliminary version

As an initial step in the validation process, we meticulously prepared preliminary versions of the scale through the method of parallel reverse translation. This involved conducting two independent translations from French to Arabic, followed by two separate translations from Arabic back into French (2 translations from French to Arabic and 2 from Arabic to French). The choice of this method is strongly supported by experts in transcultural psychology, who consider reverse translation as one of the most reliable techniques for ensuring the accuracy and cultural relevance of translated instruments. By employing parallel reverse translation, we aimed to minimize potential biases that could arise from the subjective interpretations and personal characteristics of the translators involved. In particular, Vallerand (1989) emphasizes the importance of this method, as it offers a robust safeguard against the introduction of biases during the translation process. These biases may stem from the individual linguistic preferences, cultural backgrounds, or cognitive styles of the translators, which could inadvertently influence the final translated version. By using multiple translators and comparing the outcomes, we were able to identify discrepancies and ensure a more faithful and culturally appropriate adaptation of the original scale. This meticulous approach is crucial for maintaining the validity and reliability of the instrument when applied in a new cultural context ( Vallerand, 1989).

Thus, an evaluation of the preliminary versions and preparation of an experimental version by a panel of 4 experts who evaluated the translated versions in comparison with the original French version. This evaluation was carried out on two levels: firstly, each of the items from the two French reverse translations was compared with the items from the original French version. And a second level of analysis, which the committee focused on, was the study of the technical terms used in the Arabic translations to convey the different meanings related to psychological content. According to Vallerand, the second phase of the evaluation process is crucial for assessing the quality and value of the preliminary versions of the instrument. This phase aims to refine and consolidate the translations in order to produce a single, experimental Arabic version that is both accurate and culturally appropriate. To achieve this, Vallerand advocates a committee-type approach, bringing together a group of experts capable of collectively reviewing and evaluating the draft versions. The committee, generally made up of bilingual experts in the fields concerned, plays a central role in this phase, providing a more objective and comprehensive assessment. By bringing together diverse perspectives, the committee can identify subtle nuances and potential inconsistencies in translations that might otherwise be overlooked by individual translators. This collaborative approach not only improves the accuracy of the translated instrument, but also ensures that it fits well within the cultural and linguistic context of the target population. In addition, the committee’s deliberations help resolve any ambiguities or disagreements that may have arisen during the reverse translation process. Through discussion and consensus, the committee can determine the most appropriate wording and terminology, resulting in a single, unified Arabic version that faithfully reflects the intent of the original instrument. This rigorous evaluation stage is essential to guarantee the validity and reliability of the instrument in its new cultural context ( Vallerand, 1989).

### Pre-testing the experimental questionnaire

To ensure the experimental questionnaire was suitable for its intended audience, a pre-test was conducted with 197 Tunisian health students who smoke. The purpose of this step, as highlighted by Fortin and Gagnon (2010), is multifaceted. Primarily, it aims to assess the clarity, relevance, and accessibility of the questionnaire items. This ensures that the language used is unambiguous and comprehensible, reflecting the vocabulary and cultural context of the target population.

The feedback obtained during this stage serves as a critical input for refining the tool. Specifically, participants were encouraged to suggest alternative expressions or terms for statements that appeared unclear or imprecise. By involving the target demographic directly, the pre-test phase strengthens the questionnaire’s validity and reliability. Such refinements are essential to minimize the risk of misinterpretation or response bias during the actual data collection phase.

Moreover, the involvement of committee members in approving modifications reinforces the methodological rigor of the process. Their expertise ensures that the revised items align with the study’s objectives and maintain conceptual consistency. This iterative process of testing and refinement, as emphasized by Fortin and Gagnon (2010), is a cornerstone of robust instrument development, allowing researchers to strike a balance between academic precision and participant comprehension.

In summary, pre-testing not only aids in tailoring the questionnaire to the linguistic and cultural nuances of the target group but also enhances the overall quality and effectiveness of the data collection tool.

### Reliability of the experimental version of the Q-MAT

The reliability of the Q-MAT (Questionnaire on Motivation for Abstinence from Tobacco) was evaluated through both temporal stability and internal consistency, demonstrating satisfactory results.

Firstly, the test-retest reliability yielded a correlation coefficient of 0.831 for the global score over a one-month interval. This high coefficient indicates strong temporal stability, suggesting that participants’ responses remained consistent over time. Such stability is crucial for ensuring that the questionnaire reliably captures the constructs it is designed to measure, rather than reflecting transient changes or measurement inconsistencies.

Secondly, internal consistency was assessed using Cronbach’s alpha, which produced a value of 0.840. According to Mohamad Adam et al. (2022), a Cronbach’s alpha of 0.5 is considered acceptable for basic reliability, while values between 0.70 and 0.85 are regarded as desirable, reflecting a well-balanced scale. The obtained alpha value places the Q-MAT comfortably within the desirable range, indicating that its items are appropriately interrelated and measure a cohesive construct without excessive redundancy.

The combination of these findings supports the overall reliability of the Q-MAT questionnaire. Test-retest reliability confirms its temporal stability, while internal consistency verifies the cohesiveness of its components. Together, these metrics affirm that the questionnaire is both robust and dependable for assessing the motivation for tobacco abstinence in the target population.

In addition to test-retest reliability and internal consistency, the reliability of the Q-MAT questionnaire was further assessed using the Intra-Class Correlation Coefficient (ICC). The ICC measures the agreement between test and retest scores, providing a robust indicator of the questionnaire’s stability over time. According to Fermanian (2005), the ICC is interpreted as follows: very good if ICC is 0.91 or higher, good if ICC ranges from 0.90 to 0.71, moderate if ICC is between 0.70 and 0.51, low if ICC is between 0.50 and 0.31, and very low if ICC is 0.30 or lower.

In this study, the ICC was calculated to be 0.886 with a 95% confidence interval, placing it within the “good” category. This result demonstrates strong agreement between test and retest scores, reinforcing the conclusion that the Q-MAT is a reliable instrument for measuring motivation for tobacco abstinence.

The strength of this “good” ICC value complements the findings from the test-retest correlation coefficient (0.831) and Cronbach’s alpha (0.840), all of which collectively validate the reliability of the questionnaire. Furthermore, the ICC’s ability to capture both absolute agreement and consistency between measurements makes it a critical component in reliability analysis, particularly for tools intended for repeated use in longitudinal studies or intervention evaluations.

In summary, the ICC value of 0.886 highlights a solid correlation between responses over time, indicating that the Q-MAT effectively measures stable motivational constructs. Combined with other reliability metrics, these findings provide strong evidence for the robustness of the questionnaire.

### Validity of the experimental version of the Q-MAT

The Content Validity Index (CVI) is a critical metric for evaluating how well the items of an instrument represent the full range of content relevant to the concept being measured. As described by Allen and Yen (2002), the CVI assesses the degree to which individual statements and the overall questionnaire align with the theoretical framework of the construct, ensuring comprehensive and accurate representation.

For the Q-MAT instrument, the CVI was calculated to be 0.84. This value indicates a high level of content validity, signifying that the items effectively captured the various dimensions of motivation for smoking cessation. A CVI in this range demonstrates that the instrument’s statements are well-suited to the context and concept being explored, thus supporting its relevance and appropriateness for the target population.

Achieving a CVI of 0.84 reflects the rigorous development process of the Q-MAT, including careful item formulation, expert input, and iterative refinement during pre-testing. This strong content validity reinforces the questionnaire’s credibility as a reliable and valid tool for assessing motivation to quit smoking in Tunisian health students. Furthermore, it ensures that the Q-MAT provides meaningful and actionable insights, making it a valuable resource for research and interventions in smoking cessation.

In summary, the CVI score underscores the comprehensiveness and precision of the Q-MAT instrument in addressing the construct of smoking cessation motivation, enhancing its utility in both academic and practical applications.

An analysis of the responses from 197 smoking students revealed that all questionnaire items were adequately correlated, as indicated by the Kaiser-Meyer-Olkin (KMO) test result of 0.711. This value reflects a good quality of correlations between the items and suggests that the dataset is appropriate for factor analysis. Furthermore, Bartlett’s test of sphericity yielded a statistically significant result (p < 0.001), further supporting the adequacy of the correlation matrix for factor analysis. These findings align with the criteria established by Cohen (2013), who emphasized that a significant Bartlett’s test result (p < 0.05) and a KMO index greater than 0.5 are necessary conditions to ensure the suitability of a questionnaire’s correlation matrix for dimensionality analysis.

Principal Component Analysis (PCA) is widely utilized in scale validation to assess whether individual items accurately reflect the intended constructs. In the analysis of the Q-Mat scale, the findings revealed that Item 1 explained 64.484% of the total variance, while Item 2 accounted for an additional 24.664%, together contributing to a cumulative 89.148% of the total variance. This high proportion suggests that these two components are pivotal in capturing the majority of the underlying information in the scale, specifically within the population of Tunisian health students.

The substantial variance explained by these two components underscores their critical role in the overall structure of the Q-Mat scale. This result implies that the scale is significantly influenced by these particular items, and that they play a central role in the measure of the construct. The fact that the other components show eigenvalues below 1 suggests that they add minimal additional variance, which means they contribute very little to the overall interpretation of the data. This finding aligns with the typical use of PCA, where components with eigenvalues greater than 1 are considered meaningful, while those below 1 are discarded as they offer little explanatory power.

However, it is important to contextualize these findings within the specific sample under study. Cultural and contextual differences are important factors that can influence how scale items are understood and how constructs are measured. As noted by Cokley (2015), factor structures can vary significantly across different racial or ethnic groups. In this case, the Tunisian health student population might interpret the Q-Mat items in ways that differ from other populations. For instance, sociocultural attitudes **,** educational backgrounds, and healthcare systems unique to Tunisia could shape how students respond to specific items, potentially affecting the scale’s relevance and validity.

Given that the PCA results in this specific context suggest a strong structure with two dominant components, it is crucial to recognize that these findings might not be generalizable to other populations or cultural contexts. In other settings, such as in different racial **,** ethnic **,** or educational groups, the scale might not perform as efficiently, and the distribution of explained variance across components could differ. This highlights the importance of carefully considering the cultural and contextual variables when applying or interpreting the Q-Mat scale in diverse settings.

This study represents a pioneering effort in Tunisia, being the first to translate and adapt a tool for measuring motivation to quit smoking into literary Arabic while evaluating its psychometric properties. The adaptation process was meticulously carried out to ensure cultural and linguistic relevance, thus making the tool accessible and applicable to the Tunisian context.

The translated questionnaire successfully met the criteria of the validation process, including assessments of clarity, reliability, and validity. Its psychometric properties, including internal consistency, test-retest reliability, and intra-class correlation, were satisfactory and aligned with international standards. These results demonstrate that the new instrument maintains the robustness and reliability of the original version while being tailored to the specific linguistic and cultural needs of Arabic-speaking populations.

The study’s findings are significant not only because they provide a reliable and culturally relevant tool for evaluating smoking cessation motivation in Tunisia but also because they establish a foundation for future research in the region. The availability of such a validated instrument can facilitate the development of targeted interventions and policies aimed at reducing smoking prevalence, thereby contributing to broader public health objectives.

In conclusion, this study bridges a critical gap in the field of smoking cessation research in Tunisia. The successful validation of the questionnaire highlights the importance of rigorous methodological approaches in adapting psychological tools for new contexts while preserving their original integrity and utility.

## Conclusion

The findings indicate that the Arabic version of the Q-MAT demonstrates solid reliability and validity, aligning closely with the psychometric standards of the original tool. Developing a questionnaire in Arabic to evaluate motivation for smoking cessation is of clear importance for both clinical applications and research contexts. To our knowledge, this work represents the first Tunisian initiative to render the instrument into Modern Standard Arabic and to examine its psychometric soundness. Guided by Vallerand’s cross-cultural validation methodology, the Arabic Q-MAT emerged as a trustworthy and scientifically robust measure.

## Data Availability

The datasets are not publicly available due to ethical restrictions protecting participant confidentiality. The data contain sensitive smoking behavior and personal information that could enable indirect identification despite anonymization. The Ethics Committee of Tunisia (registration number N°112, ref: CEFMS 112/2022) prohibited public data sharing as part of the ethical approval conditions, determining that open release would pose unacceptable privacy risks given the small academic community context. The underlying data supporting the results of this study are available in Zenodo at: https://doi.org/10.5281/zenodo.17179520 ( Gallas, 2025). This dataset contains CSV file including the anonymized raw data used to produce Tables 1– 3 of the manuscript, together with a file describing the variables and coding. Data are shared under the terms of the Creative Commons Attribution 4.0 International license (CC BY 4.0), which permits unrestricted use, distribution, and reproduction in any medium, provided the original authors and source are credited. Qualified researchers may request access to anonymized data from the corresponding author for legitimate research purposes. Access requires: (1) institutional ethical approval, (2) detailed research proposal, (3) signed data sharing agreement, and (4) Ethics Committee of Tunisia approval or equivalent institutional review. Access is limited to non-commercial research maintaining participant privacy protections.

## References

[ref1] Adouard V Menecier P Chapalain F : Smoking prevalence among nursing students and nursing assistants in Mâcon: 2021 survey and evolution over 13 years. *Rev. Mal. Respir.* 2022;39(4):328–333. 10.1016/j.rmr.2022.02.058 35459586

[ref2] Allen MJ Yen WM : *Introduction to measurement theory.* Waveland Press;2002.

[ref3] Aubin HJ Lagrue G Légeron P : Questionnaire Quit Smoking Motivation Questionnaire (Q-Mat): Construction and validation. *Mémoire.* 2004.

[ref4] Ben Rejeb M Abroug H Khefacha-Aissa S : Smoking behavior, knowledge, and attitudes towards anti-smoking regulations of nursing students in Sousse, Tunisia. *Rev. Epidemiol. Sante Publique.* 2016;64(2):121–127. 10.1016/j.respe.2015.12.014 26915428

[ref5] Buczkowski K Marcinowicz L Czachowski S : Motivations toward smoking cessation, reasons for relapse, and modes of quitting: Results from a qualitative study among former and current smokers. *Patient Prefer. Adherence.* 2014;8:1353–1363. 10.2147/PPA.S67767 25336926 PMC4199752

[ref6] Cohen J : *Statistical power analysis for the behavioral sciences.* Elsevier Science;2013.

[ref7] Cokley K : A confirmatory factor analysis of the Academic Motivation Scale with Black college students. *Meas. Eval. Couns. Dev.* 2015;48(2):124–139. 10.1177/0748175614563310

[ref8] Ezzaouia A Ghazzai M Smadhi H : Comportements et attitudes tabagiques chez les médecins et étudiants en médecine tunisiens. *Rev. Mal. Respir. Actual.* 2020;12(1):134. 10.1016/j.rmra.2019.11.290

[ref9] Fermanian J : Validation of assessment scales in physical medicine and rehabilitation: how are psychometric properties determined? *Ann. Rehabil. Phys. Med.* 2005;48(6):281–287. 10.1016/j.annrmp.2005.04.004 15923054

[ref10] Fortin F Gagnon J : *Fondements et étapes du processus de recherche: Méthodes quantitatives et qualitatives.* Montréal: Chenelière Éducation;2010.

[ref22] Gallas S : raw data associated with the article: Validation of the Arabic version of the smoking cessation motivation questionnaire among Tunisian student smokers.[Data set]. *Zenodo.* 2025. 10.5281/zenodo.17179520 PMC12805226 41551224

[ref11] Ghali H Ben Cheikh A Bhiri S : Therapeutic education in patients with coronary artery disease: impact on smoking cessation. *Ann. Cardiol. Angeiol.* 2022;71(4):187–193. 10.1016/j.ancard.2022.05.003 35718551

[ref12] Ghali H Rejeb OB Fredj SB : Smoking dependence and anxio-depressive disorders in Tunisian smokers attending the smoking cessation clinic in a university hospital. *J. Egypt. Public Health Assoc.* 2019;94(1):9. 10.1186/s42506-019-0009-0 32813151 PMC7364776

[ref13] Haoues M Zedini C Chadli-Chaieb M : Translation, psychometric evaluation and validation of the “Diabetes Health Profile-18” questionnaire in Arabic. *Pan Afr. Med. J.* 2021;40:212. 10.11604/pamj.2021.40.212.31410 35136475 PMC8783316

[ref14] Mohamad Adam B Hon YK Lee KY : *A step-by-step guide to questionnaire validation research.* Kuala Lumpur, Malaysia: Institute for Clinical Research, NIH MY;2022.

[ref15] Mourre ML Gurviez P : Proposition d’un modèle intégrateur de la résistance aux messages anti-tabac. *Rech. Appl. Mark.* 2015;30(3):35–62. 10.1177/0767370115571051

[ref16] Mziou E Ghali H Bhiri S : Predictive factors for successful smoking cessation in Tunisian smokers, Sousse-Tunisia: 2015–2020. *Asian Pac. J. Cancer Prev.* 2024;25(5):1615–1621. 10.31557/APJCP.2024.25.5.1615 38809633 PMC11318828

[ref17] Pougnet R Chapalain D Fortin C : Cigarette and e-cigarette use among a sample of French health students. *Rev. Mal. Respir.* 2021;38(2):164–170. 10.1016/j.rmr.2021.01.006 33583645

[ref18] Prochaska JO Diclemente CC : Toward a comprehensive model of change. *Treating Addictive Behaviors.* 1986;3–27. 10.1007/978-1-4613-2191-0_1

[ref19] Steurer J : The Delphi method: An efficient procedure to generate knowledge. *Skeletal Radiol.* 2011;40(8):959–961. 10.1007/s00256-011-1145-z 21667147

[ref20] Vallerand RJ : Vers une méthodologie de validation transculturelle de questionnaires psychologiques: Implications pour la recherche en langue française. *Can. Psychol.* 1989;30(4):662–680. 10.1037/h0079856

[ref21] Waltz CF Strickland O Lenz ER : *Measurement in nursing and health research.* 4th ed. Springer Publishing Company;2017.

