# Supplementary material for: Validation of the Arabic version of the smoking cessation motivation questionnaire among Tunisian student smokers
Source: F1000Res. 2026 Jan 13;14:1114. Originally published 2025 Oct 14. [Version 2] doi: 10.12688/f1000research.167614.2 (PMC12805226; doi:10.12688/f1000research.167614.2)
Supplement: Peer review history

# Reviewer report: Reviewer response for version 2

**DOI:** 10.5256/f1000research.192451.r449900

**Published:** 2026-01-14

**Authors:** Fithria Fithria

**Affiliations:** 1 Syiah Kuala University, Banda Aceh, Indonesia

The manuscript has been revised based on the comments. The revised manuscript is very clear and the writing is well organized.

Is the work clearly and accurately presented and does it cite the current literature?

Yes

If applicable, is the statistical analysis and its interpretation appropriate?

Yes

Are all the source data underlying the results available to ensure full reproducibility?

Yes

Is the study design appropriate and is the work technically sound?

Yes

Are the conclusions drawn adequately supported by the results?

Partly

Are sufficient details of methods and analysis provided to allow replication by others?

Yes

Reviewer Expertise:

Family Health Nursing and smoking prevention

I confirm that I have read this submission and believe that I have an appropriate level of expertise to confirm that it is of an acceptable scientific standard.

# Reviewer report: Reviewer response for version 1

**DOI:** 10.5256/f1000research.184742.r432217

**Published:** 2025-11-25

**Authors:** Fithria Fithria

**Affiliations:** 1 Syiah Kuala University, Banda Aceh, Indonesia

The manuscript will contribute significantly to future research related to smoking cessation. All sections of manuscript were written clearly and concise. However, in the background section, please add more information about the questionnaire (Q-MAT), what are the strengths of the questionnaire, why the authors interested in translating and testing that questionnaire? Then, in the conclusion section, please add study limitations and suggestions for future research related to the questionnaire (Q-MAT), especially for the researchers that also interested in translating that questionnaire into others language.

Is the work clearly and accurately presented and does it cite the current literature?

Yes

If applicable, is the statistical analysis and its interpretation appropriate?

Yes

Are all the source data underlying the results available to ensure full reproducibility?

Yes

Is the study design appropriate and is the work technically sound?

Yes

Are the conclusions drawn adequately supported by the results?

Partly

Are sufficient details of methods and analysis provided to allow replication by others?

Yes

Reviewer Expertise:

Family Health Nursing and smoking prevention

I confirm that I have read this submission and believe that I have an appropriate level of expertise to confirm that it is of an acceptable scientific standard, however I have significant reservations, as outlined above.

# Response

**Published:** 2025-11-27

**Authors:** Selma Gallas

**Affiliations:** faculty of medecine of sousse, Not Applicable, sousse, sousse, Tunisia

We sincerely thank the reviewer for their careful evaluation of our manuscript and for their constructive and valuable comments, which have greatly contributed to improving the quality and scientific rigor of our work.

**Comment 1: **In the background section, please add more information about the questionnaire (Q-MAT), its strengths, and clarify why the authors were interested in translating and testing this questionnaire.

**Response:** We appreciate this insightful suggestion. Accordingly, we have expanded the Background section to include a more detailed description of the Q-MAT questionnaire, highlighting its conceptual framework, psychometric strengths, and its relevance in assessing motivation to quit smoking. We also clarified the rationale for selecting this instrument and emphasized the importance of adapting and validating it in Arabic-speaking contexts, where culturally appropriate tools for evaluating smoking cessation motivation remain limited.

**Comment 2:** Please add study limitations and suggestions for future research related to the questionnaire (Q-MAT), especially for researchers interested in translating it into other languages.

**Response:** We thank the reviewer for this important recommendation. We have revised the Conclusion section by adding a dedicated paragraph outlining the main limitations of our study, including sample representativeness and the cross-sectional design. Furthermore, we provided suggestions for future research, encouraging longitudinal studies, validation in diverse populations, and further cross-cultural adaptations of Q-MAT into additional languages.

**Comment 3:** Are the conclusions adequately supported by the results? (Partly)

**Response:** **I**n response to this remark, we carefully reviewed and refined the Conclusion section to ensure that all statements are directly supported by the study findings. We avoided overgeneralization and reinforced alignment between results and conclusions, there by strengthening the consistency and scientific validity of the manuscript.

 Once again, we thank the reviewer for their thoughtful feedback, which has significantly enhanced the clarity and robustness of our manuscript. We believe that the revised version addresses all concerns and improves its overall quality.

# Reviewer report: Reviewer response for version 1

**DOI:** 10.5256/f1000research.184742.r424152

**Published:** 2025-11-20

**Authors:** Phindile Zifikile Shangase

**Affiliations:** 1 University of Free State, Bloemfontein, South Africa

Summary of the Article

 The current article reports on the translation, cultural adaptation, and psychometric validation of the Q-MAT into Arabic for use among Tunisian student smokers.

 The seven stages of the Vallerand’s cross-cultural validation process involve forward and backward translation, expert panel evaluation, pre-testing, and psychometric analysis, such as internal consistency, test–retest reliability, content validity, and exploratory factor analysis. This methodological cross-sectional study, which used an experimental version of the Q-MAT, was conducted among 203 smoking health science students. The respondents were asked to complete a self-report questionnaire twice, after a one-month time interval.

 A total of 197 students completed the second administration of the questionnaire, with a mean age of 18.95 ± 1.07 years and a sex ratio of 0.89. The Arabic Q-MAT showed excellent reliability: Cronbach's alpha was 0.840, reflecting good internal consistency. The test-retest reliability was also excellent, reflected by its correlation coefficient at 0.831 and intraclass correlation coefficient at 0.886. Content validity was adequate; the Content Validity Index was 0.89. Moreover, two factors were extracted using principal component analysis, accounting for 89.15% of the total variance.

 The authors conclude that the Arabic version of the Q-MAT is a psychometrically sound instrument for assessing motivation to quit smoking among young adults who speak Arabic. They recommend its application in both public health initiatives and research contexts.

 The article is clearly written, well-organized, and concise. It logically introduces the research topic, successfully connecting the public health implications of smoking to the psychological construct of motivation to quit. The rationale for translation and validation is well-presented, considering a significant gap in Arabic-language smoking cessation instruments. The background is appropriately supported by references to self-determination theory (Vallerand) and prior validations of Q-MAT in other linguistic contexts. However, the literature review could be improved by placing the Q-MAT more explicitly in the context of similar instruments, such as the Motivation to Stop Scale, the Readiness to Quit Ladder, and stages-of-change measures. This would further strengthen the theoretical rationale for selecting the Q-MAT as the instrument of choice. On the whole, the presentation is clear, the argument coherent, and citations adequate though somewhat limited in scope.

 The authors adopt a cross-sectional psychometric validation design, which is well-suited to the study’s objectives. The seven-step translation and adaptation process, including forward translation, back translation, expert review, pilot testing, and validation aligns with internationally recognised standards, such as those outlined in the World Health Organization (WHO) guidelines. The use of a sample comprising approximately 200 student smokers (203 in the initial administration and 197 in the follow-up) provides sufficient statistical power for conducting factor analysis and reliability assessments. Overall, the methodological approach is robust and appropriately implemented for an initial validation study. However, a notable limitation is the homogeneity of the sample, consisting solely of young health science students, which may constrain the external validity and generalisability of the findings. While the authors acknowledge this limitation, further elaboration on its implications for broader population applicability would strengthen the discussion.

 The translation process, inclusion/exclusion criteria, demographic data, and procedures of the psychometric testing were reasonably described. Reliability and validity analyses were presented clearly and reproducible, specifying the statistical software/criteria used. To enhance replicability, the authors are encouraged to present the final Arabic items either within the text or as a supplementary table, highlighting any modification made to the original scale.

 The authors have provided a Data Availability Statement with open access to the anonymized dataset and supplemental files.

 Conclusions are indeed consistent with the data presented: the Arabic Q-MAT demonstrates a high degree of internal consistency, good temporal stability, and acceptable construct validity. The authors avoid overgeneralization beyond their population of interest, namely, Tunisian student smokers, and therefore adequately refer to their findings as preliminary validation.

 They correctly acknowledge limitations such as sample specificity, absence of CFA, and limited external validity, recommending further testing in other Arabic populations. This balanced interpretation supports the credibility of their conclusions.

 The data presented fully support the authors' conclusions. The Arabic Q-MAT version has high internal consistency, satisfactory temporal stability, and acceptable construct validity. What is important is that the authors have been cautious in making any generalizations across populations, and therefore, they limit their claims to Tunisian student smokers and characterize their findings as preliminary validation.

 They further provide a clear account of the study's limitations, such as the specificity of the sample, the absence of CFA, and the restricted external validity. Their recommendation for further validation across diverse Arabic-speaking populations reflects a balanced and responsible interpretation of the results, thereby enhancing the credibility of their conclusions.

 The article presents a methodologically rigorous and transparent validation study that makes a valuable contribution to the development of culturally adapted Arabic-language psychometric instruments for tobacco cessation research.

 Minor suggestions for authors: In the socio-economic status category, a total of 218 responses are recorded while the actual number of participants is 203; the researchers should check this inconsistency and give a reason for this variation or adjust the data to ensure accuracy. Overall, the present study was an important and well-conducted contribution to Arabic language behavioral health research, strengthening the methodological basis for culturally appropriate psychometric assessment in smoking cessation contexts.

Is the work clearly and accurately presented and does it cite the current literature?

Yes

If applicable, is the statistical analysis and its interpretation appropriate?

I cannot comment. A qualified statistician is required.

Are all the source data underlying the results available to ensure full reproducibility?

Yes

Is the study design appropriate and is the work technically sound?

Yes

Are the conclusions drawn adequately supported by the results?

Yes

Are sufficient details of methods and analysis provided to allow replication by others?

Yes

Reviewer Expertise:

Public Health

I confirm that I have read this submission and believe that I have an appropriate level of expertise to confirm that it is of an acceptable scientific standard.

# Response

**Published:** 2025-11-20

**Authors:** Selma Gallas

**Affiliations:** faculty of medecine of sousse, Not Applicable, sousse, sousse, Tunisia

Dear Reviewer, Phindile Zifikile Shangase,

 We sincerely thank you for your thorough, rigorous, and constructive evaluation of our manuscript, as well as for your complimentary comments regarding the methodological quality and clarity of our work. Your comments have contributed significantly to the improvement of our article.

 ** 218 responses were recorded, while the number of participants is 203.

**Response:** We thank the reviewer for pointing out this inconsistency. After verification, this was due to a data entry error. The data has been corrected and harmonized, and the corresponding table will be updated to accurately reflect the actual number of participants (n = 203). And an explanatory note will be added to the results section.

**Conclusion**

 We would like to warmly thank the reviewer for the quality of their expertise and constructive comments, which have helped to improve the scientific rigor, clarity, and scope of our work. We hope that the revisions made fully address their recommendations.

 We remain at your disposal for any further clarification.

 Yours sincerely,

 Selma Gallas
